# Acute Effects of Exergaming on Students’ Executive Functions and Situational Interest in Elementary Physical Education

**DOI:** 10.3390/ijerph20031902

**Published:** 2023-01-20

**Authors:** Athanasios Kolovelonis, Marina Papastergiou, Evdoxia Samara, Marios Goudas

**Affiliations:** Department of Physical Education and Sport Science, University of Thessaly, 42100 Trikala, Greece

**Keywords:** exergames, inhibition, cognitive flexibility, design fluency

## Abstract

Two studies were conducted to examine the acute effects of exergaming on students’ executive functions and to explore their situational interest regarding these games in elementary physical education. The first study involved a two-group, repeated measures, cross-over quasi-experimental design. Participants were 74 (36 boys) fourth- and fifth-grade students who were assigned to the experimental (38 students) and the waiting list control (36 students) group. The single physical education session with exergames was first implemented with the initial experimental group and after the post-test, the waiting list control group received the intervention. In the second study, a pre-test post-test, within-subjects design was involved with the experimental group students (48 fourth- and fifth-grade students, 27 boys) who participated in a booster single physical education session with exergames two months after their involvement in a four-week intervention with cognitively challenging physical activity games. Both studies involved pre- and post-intervention measures for executive functions using the design fluency test and a post-test measure for situational interest. During the acute session, students had to follow the movements of an on-screen dancing character in time to a chosen song of the Just Dance 2015 exergame. The results of the first study showed that experimental group students improved significantly from pre- to post-test their scores in design fluency and in cognitive flexibility and in the total score of the design fluency test and their improvements were higher compared to the waiting list control group. The waiting list control group students, after receiving the acute session with exergames, significantly improved their scores in design fluency, inhibition, and cognitive flexibility and in the total score of the design fluency test compared to their pre-intervention scores. Moreover, the second study showed that students’ total score in the design fluency test improved significantly from pre- to post-intervention. In both studies, students reported generally high scores in all subscales of the situational interest questionnaire. These results suggested that an acute exergame-based physical education session attracted students’ interest and positively triggered their executive functions.

## 1. Introduction

Exergames, a term derived from the combination of “exercise” and “games”, are electronic games that demand gross body movements [1]. As opposed to sedentary electronic games, which mainly require finger and hand movements, exergames demand substantial physical activity (e.g., lower-limb or whole-body movements) for interacting with the game environment and rely on various exertion interfaces (e.g., dance pads, bicycle ergometers) or motion tracking technologies [2,3]. The first popular, cost-effective exergame has been Dance Dance Revolution (DDR) [1,4], which was released in 1998 by Konami [5]. In DDR, the player has to dance by stepping on the multi-directional sensor arrows of a dance pad placed on the floor in synchronization with music, following on-screen arrows that correspond to the arrows of the dance pad [1,2,3].

Various companies have produced motion sensing devices for exergaming [4], such as cameras or controllers that detect the player’s movement, which is transferred to the game environment and displayed on-screen as a movement of a character or of an avatar representing the player [1,6]. The release of the Wii gaming console, the Wii Remote controller, and the Wii Balance Boar, a device that tracked the player’s center of balance, together with the Wii Sports and Wii Fit exergames, in 2006–2007 by Nintendo, revolutionized exergaming enabling players to perform sports (e.g., tennis) movements, yoga, strength, aerobics and balance training using the controller and/or the balance board [7,8]. In 2010, Sony released the PlayStation Move controller for its PlayStation console [9], and Microsoft launched Kinect, a device with motion-sensing cameras that enabled players to freely interact with games through body movements without any controller [4], for its Xbox console, together with the Kinect Sports exergames [10]. These advances further spurred exergame development.

Various studies have shown that playing exergames, such as DDR, Nintendo Wii Sports, or Xbox Kinect dance and sports games, elicits energy expenditure comparable to moderate-intensity physical activity in primary school children [11,12,13]. Playing exergames, such as Nintendo Wii Fit, Xbox Kinect Sports, or other dance, fitness, and yoga games, regularly at school, was also found to increase school day energy expenditure [14,15]. Exergames were also found to improve the fundamental motor skills [16] and fitness [17] of primary school children. A recent overview of 17 systematic reviews and meta-analyses [18] showed that exergames, such as dance, aerobics, and sports games, positively impacted both the physical functioning and the mental health of children.

Regarding motivational variables, many studies converge that exergaming elicits high levels of enjoyment in children [13,14,17,19,20,21]. Furthermore, exergaming has been found to improve children’s attitudes toward physical activity, in terms of self-efficacy, intention to exercise, and perceived social support to exercise [15,17]. Apart from physical benefits, Staiano and Calvert [1] have argued that exergames may also entail psychosocial benefits (e.g., increased social interaction through multiplayer modes) and cognitive benefits (e.g., improved executive functions).

Executive functions are a family of higher-order cognitive processes that facilitate goal-directed behavior and cognitive flexibility. These processes are necessary for students to deal with novel, challenging, and complex conditions in learning environments. The three core executive functions identified in the literature are inhibition, working memory, and cognitive flexibility [22]. Inhibition helps students to control their behavior, thoughts, or emotions in order to act properly in changing learning environments. Working memory works as a short-term storage of information that students can use or handle during their involvement in learning conditions. Cognitive flexibility helps students to be flexible when they face new demands, rules, or priorities in learning environments and have to shift their attention between task demands or use a new approach for solving a problem.

The importance of executive functions has been highlighted not only due to their obvious implications for students’ development but also due to their practical implications for promoting learning and performance. Indeed, executive functions are positively associated with health, quality of life, and success in school and life [23]. Furthermore, they are important for school readiness [24], academic achievement [25], and motor ability [26]. Executive functions may also facilitate self-regulation and metacognitive control [27], which in turn lead to positive outcomes such as improved motor and sports performance, satisfaction, and enjoyment [28,29].

Physical activity may have positive effects on students’ executive functions. Indeed, recent reviews and meta-analyses provided evidence of the beneficial effects of long-term physical activity interventions on students’ executive functions [30,31]. For example, a cognitively enriched sports program had positive effects on students’ working memory and cognitive flexibility [32] while a six-month football intervention improved students’ executive function compared to a passive control group [33]. However, other research evidence has suggested that executive functions were not affected by physical activity [34,35]. Furthermore, acute physical activity may have positive effects on students’ executive functions, although mixed results have also been reported [36].

Sports and physical activities vary in their characteristics and in the degree of involving students in cognitively challenging conditions [37]. Thus, the quality and the quantity of physical activity seem to play a role in the inconsistent finding regarding the effects of physical activity on executive functions [38]. Interestingly, the largest review regarding executive functions has suggested that not all types of physical activity can effectively affect executive functions [39]. This means that appropriate content should be used for triggering students’ executive functions. The results of the abovementioned reviews [30,31] showed that the larger effects on students’ executive functions resulted from physical activities enriched with cognitive challenges. For example, involving students in unpredictable and complex conditions requiring alternating between action plans or overriding prior actions and acting in a totally different way [40] and in novel, challenging, and not highly repetitive or automatized tasks [41] is considered appropriate for developing students’ executive functions. In this line, recent research in physical education examined the effects of cognitively challenging physical activity games on students’ executive functions. In particular, Kolovelonis and Goudas [42] found that three different types of these games based on a different principle of mental engagement (contextual interference, mental control, or discovery) were equally effective in triggering students’ executive functions. Moreover, students who played these games improved their executive functions more than students who were taught soccer or track and field skills [43] or students who practiced activities for enhancing their health-related fitness components [44]. Expanding these acute experiments, Kolovelonis et al. [45] found that a four-week intervention in physical education involving cognitively challenging physical activity games had also positive effects on students’ executive functions.

The abovementioned evidence suggests that appropriately designed activities in physical education may effectively trigger students’ executive functions. Exergames may also fall into this category. However, research evidence regarding the effects of exergames on students’ executive functions is generally limited. Indeed, although numerous studies have addressed the impact of exergames on the executive functions of adults, and especially older ones, generally showing positive effects [46,47,48], research in school-age children is still comparatively limited and, more specifically, research in school settings and real classroom situations is even scarcer.

Indeed, very few studies have been conducted in children’s populations showing a positive impact of acute or long-term exergaming on children’s executive functions. In a study by Best [49], 33 children, aged 6 to 10 years, participated in four conditions of equal duration (20 min): physically and cognitively engaging exergaming, physically but not cognitively engaging exergaming, cognitively but not physically engaging sedentary video gaming and not physically nor cognitively engaging video activity (watching video). Exergaming (either cognitively demanding or not) was found to improve children’s executive functions, which led to the conclusion that it is the physical challenge of exergames that may enhance executive functioning. In another study [50], 147 children, aged 7 to 12 years, were assigned to four conditions of equal duration (20 min): physically and cognitively engaging activity (playing a dance-based exergame that resembled DDR), physically but not cognitively engaging activity (aerobic exercise), cognitively but not physically engaging activity (sedentary playing the dance-based exergame through keyboard/mouse) and not physically nor cognitively engaging activity (sitting and chatting). The children that had played the dance-based game (either actually dancing or sedentarily) improved their executive functions (accuracy on the switch-task Flanker Test and reaction time on the standard Flanker Test) more than those of the other two conditions, which led to the conclusion that it is the cognitive challenge of exergames that may benefit executive functioning. The two aforementioned studies were conducted in researchers’ settings and in an after-school program, as it is mentioned in or can be deduced from the corresponding papers. In a study conducted on 94 children and adolescents, aged 10 to 16 years, who were attending a summer camp [51], the participants that had played Nintendo Wii Fit aerobics and balance exergames during five 30 min sessions, distributed over five weeks, enjoyed the experience, and improved their executive functions from pretest to posttest, whereas those who had played a less active exergame and those who had participated in regular camp sports activities had no improvement. Finally, in a study that was conducted in a school setting, the sample of which, however, were not children but 65 adolescent boys, aged 13–16 years [52], participants were randomly assigned to one of three conditions of equal duration (15 min): playing a physically and cognitively challenging exergame (Shape Up by Ubisoft), playing a purely physically challenging running exergame, and passively watching a video regarding running. The first group (Shape Up) was found to perform significantly better in cognitive flexibility compared to the other two groups.

Moreover, successful physical education programs should involve tasks, activities, and games that attract students’ interest and increase their motivation to participate in the lesson. Situational interest may reflect students’ recognition of the appealing features of tasks and activities and thus may be considered as an important motivational variable in physical education [53]. Situational interest is context-specific and environmentally activated and has been conceptualized as a multidimensional construct. In particular, encompass five dimensions: novelty, instant enjoyment, exploration intention, attention demand, and challenge [53]. Novelty reflects what is known and unknown. Instant enjoyment is associated with positive feelings of satisfaction after participating in an activity. Exploration intention reflects the aspects of an activity that drive learners to explore or discover. Attention demand refers to the mental effort required by an activity to be involved. Challenge reflects the level of difficulty of an activity relative to one’s ability [54]. An overall total interest element has also been identified referring to students’ overall assessment of situational interest [53]. Thus, situational interest is increased when students participate in tasks they perceive as new, challenging, demanding, or allowing them to explore possibilities and offer enjoyment.

Research evidence has suggested that exergames may positively affect students’ situational interest [19]. In particular, in a laboratory study, Roure et al. [55] found that a bike exergame had positive effects on undergraduate students’ health-related physical activity outcomes and situational interest. Sun [56] found that elementary students who participated in exergaming reported higher levels of situational interest than students who involved in a cardiovascular fitness unit. However, students’ situational interest declined significantly in both groups and at the same rate at the end of the 4-week intervention. A similar decline in students’ situational interest was also found in a follow-up study [57]. Moreover, Sun and Gao [58] found that elementary students who were stepping during playing an educational video game reported higher levels of situational interest than their counterparts in the control group who simply played the video game. These findings provide preliminary evidence suggesting that exergames may positively affect students’ situational interests. However, further research is needed, especially in real life physical education conditions to explore the motivational power of exergaming.

### The Present Study

As deduced from the above review of the research literature and to the best of the authors’ knowledge, no study thus far has investigated the feasibility and the effectiveness of using exergaming as a means for executive function development in children belonging to the general population, in realistic classroom situations, that are inherently social and where the available technological resources may be more constrained than in laboratory settings. The studies presented in this paper attempt to fill this gap in the research literature. The interventions were conducted within the framework of the physical education course of a Greek primary school and focused on the acute effects of exergaming.

Quality physical education should focus on students’ holistic development including cognitive, emotional, social, and organic aspects, promoting healthy lifestyles including lifelong participation in sports and physical activity, and enhancing their well-being and quality of life. Moreover, providing students with positive, challenging, and developmentally appropriate learning experiences in physical education may help them to acquire the necessary knowledge, skills, attitudes, and values for leading a physically active life, now and in the future [59].

In this line, selecting physical education content that focuses on multiple aspects of students’ development is warranted. Indeed, recent views regarding the role of physical activity programs have highlighted the dual role that these programs should serve. That is, to increase not only students’ physical activity but also to promote their cognitive development [60]. This can be completed with the implementation of appropriately designed cognitively enriched physical activity programs that promote students’ physical activity and trigger their executive functions. Considering, however, that not all types of physical activity programs can serve this role [39], further research is needed in this field. Exergames may be considered appropriate means of increasing students’ physical activity and triggering their executive functions during physical education, especially on certain occasions when adaptations to regular programs are needed such as, for example, on rainy days. However, as already mentioned, exergaming seems to increase students’ physical activity, but little is known regarding its effects on students’ executive functions. Furthermore, students’ situational interest regarding exergames should be further explored.

In this line, two studies were conducted including one acute experiment each to examine the effects of exergaming on students’ executive functions and to explore students’ situational interest regarding these games. The research questions that guided these studies were:

(a) Can an exergames activity within physical education increase students’ executive functions (Study 1)?
(b) Can an exergames activity within physical education have a booster effect on the executive functions of students who had already participated in a respective intervention increasing their executive functions (Study 2)?
(c) What are students’ levels of situational interest after participating in the single physical education session with exergames (Study 1 and 2)?

It was hypothesized that participation in the single physical education session with exergames would increase students’ executive functions from pre- to post-test. Moreover, it was expected that students would report high scores in all subscales of the situational interest questionnaire after the intervention. No hypothesis regarding the second research question was set due to a lack of previous evidence.

## 2. Materials and Methods

### 2.1. Design

In the first study, a two-group, repeated measures, cross-over quasi-experimental design was used. The single physical education session with exergames was first implemented with the initial experimental group and after the post-test, the waiting list control group received the intervention. The design involved pre- and post-intervention measures for executive functions and a post-test measure for situational interest. In the second study, a pre-test post-test, and within-subjects design were involved. Experimental group students participated in a single physical education session with exergames two months after the completion of a four-week intervention with cognitively challenging physical activity games [45]. The design involved pre- and post-intervention measures for executive functions and a post-test measure for situational interest.

### 2.2. Participants and Settings

A total number of 122 (*M*age = 9.98 years old, *SD* = 0.59, 63 boys) fourth- (61 students, *M*age = 9.46 years old, *SD* = 0.24, 31 boys) and fifth-grade (61 students, *M*age = 10.12 years old, *SD* = 0.56, 32 boys) students participated in the experiments of the two studies. In particular, in the first study, participants were 35 (15 boys) fourth- and 39 (21 boys) fifth-grade students. These students had no previous experience with exergames and attended two fourth- and two fifth-grade classes from one elementary school and they were assigned to the experimental (17 fourth- and 21 fifth-grade students) and the control (18 fourth- and 18 fifth-grade students) group. In the second study, participants were 26 (16 boys) fourth- and 22 (11 boys) fifth-grade students who had been previously enrolled in a larger intervention program [45]. These students had some previous experience with exergames and attended one fourth- and one fifth-grade class from one elementary school.

Physical education in Greece is delivered by specialized physical education teachers in coeducational classes. It is mandatory and includes three 45 min sessions per week for fourth-grade students and two 45 min sessions per week for fifth-grade students. The central aim of physical education is to promote students’ lifelong physical activity and to improve the quality of their lives.

### 2.3. Measures

#### 2.3.1. The Design Fluency Test

For measuring students’ executive functions, the design fluency (DF) test was used [61]. This test asks students to draw as many different designs as possible by connecting dots with a pencil using four consecutive straight lines. Each one of the three conditions of the test lasted one minute, and the response sheet included square boxes with unstructured arrays of dots. In particular, in condition 1 measuring design fluency, students had to draw designs in boxes containing five solid dots using four consecutive straight lines. In condition 2, measuring inhibition, boxes contained five solid and five blank dots, and students had to draw designs connecting only blank dots. In condition 3, measuring cognitive flexibility, boxes contained five solid and five blank dots, and students had to draw designs alternating between connecting a solid and a blank dot. They could start either from a solid or a blank dot. Students’ score in each condition of the test was the number of correct and unique designs. A total score combining the scores in the three conditions was also calculated [61].

#### 2.3.2. Situational Interest

Students’ situational interest during the physical education session was measured with the situational interest scale [62]. This scale measures five situational interest dimensions (consisting of three items each): novelty (e.g., “what we did today was new to me”); instant enjoyment (e.g., “What we were learning inspires me to try out what we were learning”); exploration intention (e.g., “I wanted to know more about how to do what we were learning today”); attention demand (e.g., “I was focused on what we were learning today”); and challenge (e.g., “What we were learning was complex”). A total interest subscale (four items) was also included (e.g., “What we were learning was interesting for me to do”). All items were rated on a five-point Likert scale ranging from 1 (strongly disagree) to 5 (strongly agree). This situational interest scale has been adapted in the Greek language [42] demonstrating a good model fit of the sixth-factor solution, *χ^2^* (137) = 159.82, *p* = 0.088, *χ^2^*/*df* = 1.17, *NNFI* = 0.980, *CFI* = 0.984, *RMSEA* = 0.034 (90% CI: 0.000–0.055). For Study 1, Cronbach’s alpha for the subscales was: total interest, 0.84, instant enjoyment, 0.83, exploration, 0.76, attention demand, 0.60, challenge, 0.55, and novelty, 0.90. Due to low internal consistency of the attention demand and the challenge subscales, these subscales were excluded. For Study 2, Cronbach’s alphas for the respective subscales were: total interest, 0.84, instant enjoyment, 0.87, exploration, 0.69, and novelty, 0.89.

### 2.4. Procedures

The University Ethics Review Committee and the Ministry of Education provided ethical approval for conducting the study (1522, 5 June 2019). School principals and physical education teachers provided their permissions too. Participation in the studies was voluntary for students and the only inclusion criterion was to provide parental written consent. Both acute experiments were implemented by an experimenter blind to the aims of the studies, with a master’s degree in physical education. Procedures of the experiments were piloted with classes of students not participating in this study and modifications were applied if necessary. These procedures were identical in both studies and are described in detail next. In particular, the pre-test measure for the design fluency test was administered one week before the acute experiment. Students completed the test in their classroom at the beginning of the first physical education session of the week. They were provided with instructions regarding the completion of each condition of the test, observed the experimenter perform one trial on the classroom blackboard, and performed a trial including three boxes of dots. The post-test measure for design fluency and situational interest was administered just after the end of the acute experiment.

### 2.5. Description of the Intervention

The Just Dance 2015 exergame by Ubisoft [63] was used on a Microsoft Xbox One console equipped with Kinect. The game is rated “3” by the Pan European Game Information (PEGI), namely it is suitable for all age groups [64]. It comprises 43 popular songs [65] and can accommodate up to 6 simultaneous local players (local multiplayer mode) [63]. The aim of the game is to follow the movements of an on-screen dancing character in time to a chosen song [65,66]. Each player’s movement is captured by Kinect and, while the player is dancing to the song, he/she receives immediate, continuous feedback in the form of visual cues regarding his/her performance (namely, “OK”, “Good” and “Perfect”, if the performance is just acceptable, good, or perfect, respectively). After the song has finished, each player’s scoring points for the specific song are displayed, which are based on how accurately the player has mimicked the character’s movements while he/she was dancing the song [66,67].

Due to the unavailability of multiple videogaming consoles, this study was performed with minimum resources (namely one console), which differentiates it from prior studies [51] that made use of more equipment (e.g., 8 consoles). Furthermore, extending relevant prior research that had examined solitary or paired exergaming [51,66,68,69], in this study the local multiplayer mode of the game was adopted to serve the class condition.

For the first study, the experiment took place in the school event hall, which occasionally serves as a gym during rainy days. A 32-inch TV, which was available in the school, was connected to the Xbox One console through an HDMI cable and served as a video and audio output device. For the second study, the experiment took place in the school computer lab. The overhead projector and its speakers, that was already installed in the computer lab, were connected to the Xbox One console through an HDMI cable and served as a video and audio output device.

In both studies, all students were simultaneously physically active during exergames activity. In particular, in the first study, children entered the hall in whole classes and received instructions. They were then divided into teams of 6 persons each, and the 36-person teams were placed in rows. The first 6-person team was placed in the front row, facing the TV monitor and Kinect so that the dance movements of those 6 children could be tracked by Kinect and their performances are commented (“OK”, “Good”, “Perfect”) and scored within the game environment. The second and the third 6-person teams were placed in the second and third row, respectively (behind the first team), and could dance following the on-screen character’s movements, without however being tracked and scored. After the first 6-person team had danced, it moved to the last row, and the second team moved to the front row. Rotating in this way, every team had the opportunity to dance in the front row with tracking and also to dance following the on-screen character while being in the back rows.

Taking into account prior relevant research that had shown that both solitary [66] and competitive [69] exergame play are more effective than cooperative exergaming in enhancing executive skills, in this study that was conducted at class level, the children within each 6-player team (those placed in the first row) were not asked to cooperate to achieve a higher combined score. However, they were neither asked to compete against each other, to who will achieve the higher individual score, given that, in competitive exergaming, non-competitive children might feel pressed [70]. Nevertheless, the fact that after each song, the individual scores that the 6 players had achieved were displayed on-screen, inevitably added a competitive flavor to exergame play. However, these scores were not used for evaluating or ranking students’ performance, rather all students were prompted to do their best and their efforts were reinforced.

The children danced to two songs. First, they danced to the song “Happy” (released in 2013 by Pharrell Williams), which lasts 2 min and 52 s, and is deemed to be of moderate difficulty requiring moderate effort on the part of the dancer [71]. Due to the rotation of the six-person teams, each child danced the song three times but was tracked and received feedback once. In continuation, the children danced to the song “Love Me Again” (released in 2013 by John Newman), which lasts 3 min and 35 s and is also considered of moderate difficulty [72]. Total dance time for each child was 19 and a half minutes. In the second study, due to space restrictions, students entered the computer lab in groups of eight and formed two rows of four with the first one being tracked and the second dancing without tracking. The same songs as in the first study were used. Total dancing time for each child was about 13 min. For the first study, total duration of exergame play was similar to the duration reported in previous relevant studies [49,50,52,66,73,74] while, for the second study, total duration was somewhat lower (54: 20 min, 55: 15 min, 53: 20 min, 67: 20 min).

### 2.6. Statistical Analysis

Descriptive statistics including mean scores and standard deviations were calculated for students’ scores in the three conditions and the total score of the DF test and the four subscales of the situational interest questionnaire. Data from study 1 were analyzed through a 2 (Group) X 2 (Time) repeated measures MANOVA with group as independent factor, time as the repeated factor and students’ scores in the three conditions of the DF test as the dependent variables. Univariate tests for each test condition and paired sample t-tests for examining pre- to post-test differences followed. Moreover, a 2 (Group) X 2 (Time) repeated measures ANOVA with students’ total score in the DF test as the dependent variable was used. The effect of the exergames session on waitlist control group students’ executive functions was examined through a repeated measures MANOVA with time as the repeated factor (Time 2 and Time 3) and students’ scores in the three conditions of the DF test at Time 2 and Time 3 as dependent variables. A repeated measures ANOVA with students’ total score in the DF test as the dependent variable was also conducted. Data from study 2 were analyzed through a repeated measures MANOVA with time (pre-test and post-test) as the repeated factor and students’ scores in the three conditions of the DF test as dependent variables. Moreover, a paired samples t-test was used for comparing students’ total scores in the DF test between pre- and post-test. Effects sizes of partial η^2^ and Cohen’s *d* were also calculated [75].

## 3. Results

### 3.1. Preliminary Analyses

A principal components analysis with varimax rotation was performed on the scores of the three conditions of the design fluency test (Study 1). The Kaiser–Meyer–Olkin (0.63) and the Bartlett test of sphericity, *χ*^2^ (3) = 169.7, *p* < 0.001, confirmed the sampling adequacy and the appropriateness of the correlation matrix for this analysis. One principal component with eigenvalue >1 resulted in explaining 64.2 of the variances. Therefore, in subsequent analyses, both the scores of the three test conditions and their total score were used. An ANOVA showed that boys and girls did not differ on the total DF score, *F* (1, 71) = 0.82, *p* = 0.37, while a MANOVA showed that their difference on the three DF conditions was not significant, *F* (3, 69) = 0.38, *p* = 0.70. An ANOVA showed that the two grade groups did not differ on the total DF score, *F* (1, 71) = 0.52, *p* = 0.48 while a MANOVA showed that they did not differ on the three DF conditions, *F* (3, 69) = 0.17, *p* = 0.92. Thus, scores of both genders and grades were collapsed within the experimental and waitlist control groups. The experimental and the control group did not differ at Time 1, neither on the three DF test conditions, *F* (3, 69) = 1.12, *p* = 0.35, nor on the total score of the DF test, *F* (1, 71) = 0.05, *p* = 0.83.

### 3.2. Study 1

#### 3.2.1. Effect of the Exergames on Experimental Group Students’ Executive Function

Table 1 presents descriptive statistics for the executive functions test scores separately for the experimental and the waitlist control group and for the three time measures. A 2 (Group) X 2 (Time) repeated measures MANOVA with the three test condition scores as dependent variables, showed a significant Group X Time interaction, *F* (3, 66) = 5.76, *p* < 0.001, partial η^2^ = 0.21, power = 0.94. Subsequent univariate tests showed a significant Group X Time interaction for condition 1 test scores, *F* (1, 68) = 15.4, *p* < 0.001, partial η^2^ = 0.19, power = 0.97, a non-significant Group X Time interaction for condition 2 test scores *F* (1, 68) = 0.38, *p* = 0.54, and a marginally significant Group X Time interaction for test condition 3 scores *F* (1, 68) = 4.17, *p* = 0.045, partial η^2^ = 0.06, power = 0.52. Paired sample t-tests showed that both the experimental [*t* (33) = −6.0, *p* < 0.001, *d* = 1.12, for test condition 1 scores, and *t* (33) = −3.7, *p* < 0.001, *d* = 0.62, for test condition 3 scores] and the waitlist control group [*t* (35) = −4,2, *p* < 0.001, *d* = 0.56, for test condition 1 scores, and *t* (35) = −2.36, *p* = 0.024, *d* = 0.32 for test condition 3 scores] scored higher at Time 2, however, the improvement was significantly higher for the experimental group. Regarding the total DF score, the 2 (Group) X 2 (Time) repeated measures ANOVA showed a significant Group X Time interaction, *F* (1, 68) = 12.66, *p* < 0.001, partial η2 = 0.16, power = 0.94. Paired sample t-tests showed that both the experimental, *t* (33) = −6.7, *p* < 0.001, *d* = 0.92, and the waitlist control group, *t* (36) = −5.0, *p* < 0.001, *d* = 0.64 improved, however, the improvement was higher for the experimental group.

#### 3.2.2. Effect of the Exergames Session on Waitlist Control Group Students’ Executive Functions

A repeated measures ANOVA with the total DF score as the dependent variable and measures at Time 2 and at Time 3, showed that the waitlist control group students improved significantly after receiving the exergames session, *F* (1, 35) = 48.76, *p* < 0.001, partial η^2^ = 0.58, power = 1.0. Similarly, a repeated measures MANOVA with the three conditions scores of the DF test measures at Time 2 and Time 3, showed a multivariate significant effect for time, *F* (3, 33) = 17.55, partial η^2^ = 0.62, power = 1.0. Subsequent univariate tests showed an improvement in all three test condition scores, DF condition 1: *F* (1, 35) = 20.1, *p* < 0.001, partial η^2^ = 0.36, power = 0.99, DF condition 2: *F* (1, 35) = 31.22, *p* < 0.001, partial η^2^ = 47, power = 1.0, DF condition 3: *F* (1, 35) = 32.68, *p* < 0.001, partial η^2^ = 48, power = 1.0.

#### 3.2.3. Students’ Situational Interest in Exergames Sessions

Table 2 presents descriptive statistics for the four subscales of situational interest separately for the experimental and the waitlist control group in Study 1 and for the experimental group in Study 2. Generally, in both studies, students reported high scores (all above the mid-point of the five-point scale) in the four subscales of the situational interest questionnaire.

### 3.3. Study 2

Table 3 presents descriptive statistics for students’ executive function test scores before and after the exergames session. A paired samples t-test with the total DF scores before and after the session provided a significant result, *t* (38) = −2.44, *p* = 0.019, *d* = 0.27. However, a repeated measures MANOVA with the three DF conditions scores as the dependent variables provided a non-significant result, *F* (3, 36) = 1.94, *p* = 0.14.

## 4. Discussion

This study included two experiments to examine the acute effects of exergaming on students’ executive functions and situational interests. Generally, the results of these experiments provided supporting evidence regarding the positive effects of exergaming on students’ executive functions in physical education. Moreover, students reported generally high scores in situational interest questionnaire subscales. These results are discussed next in detail with reference to previous findings and to theoretical and practical implications for promoting students’ executive functions in physical education and the potential contribution of exergames in this direction.

Students who participated in the acute physical education session with exergames improved significantly from pre- to post-test their scores in the first and the third conditions and in the total score of the design fluency test. Moreover, these improvements from the pre- to post-test were higher for the experimental group compared to the waitlist control group. Similarly, the waitlist control group students significantly improved their scores in the three conditions and in the total score of the design fluency test after receiving the acute session with exergames compared to their pre-intervention scores. In sum, these results suggest that an acute physical education session with exergames can positively trigger students’ executive functions. These results are consistent with previous findings suggesting that exergames can have positive effects on students’ executive functions [49,50,51]. Moreover, this study expanded the previous ones by showing that students’ executive functions can be improved by a school-based intervention incorporated into the regular physical education schedule. The present results are also consistent with previous findings suggesting that cognitively enriched physical education programs, such as those including cognitively challenging physical activity games, can effectively promote students’ executive functions [43,44,45]. Thus, exergames may be considered appropriate content for triggering students’ executive functions [39].

An interesting variation in these results was that in the first study, the improvement from pre- to post-test in students’ scores on inhibition did not differ between the experimental and the waitlist control group. This result seems to contrast previous evidence showing that inhibition is the aspect of executive functions that mostly benefited from physical activity interventions involving cognitively enriched activities [31]. However, a similar finding was also reported by Benzing et al. [52] showing positive effects of an acute bout of exergame-based physical activity on seventh- to ninth-grade students’ cognitive flexibility (measured with the design fluency test) but not on inhibition. Future research should further examine this issue exploring the effects of exergames on all aspects of executive functions including working memory, which was not examined in this study.

Regarding the booster effects of exergames, the results of the second study showed that students’ total scores in the design fluency test improved significantly after the acute session with the exergames compared to the pre-session scores. However, students’ scores in the three conditions of the design fluency tests improved from pre- to post-test but this improvement was not significant. Students in the second experiment had already participated in a four-week intervention and their executive functions had been significantly increased after the intervention and retained higher two months later compared to the pre-intervention scores [38]. Under these conditions, this small improvement on students’ total score in the executive functions test after the acute experiment with exergames can be considered important.

Potential mechanisms explaining the effects of exergames on students’ executive functions may be looked into the nature of these games. In particular, recent reviews [30,31] have suggested that executive functions are optimally developed when students are involved in physical activities enriched with cognitive challenges. In the case of exergames and especially dance exergames, such as the one utilized in the present research, the fact that, apart from whole body activation, these games also require learning and imitation of sequences of complex movements (instead of the automatic replication of simple, segmented movements), a task which demands attention, memory, coordination, and speed of action [50,52,66,73,74,76], may account for their beneficial effects on executive functions.

Regarding situational interest, the results of this study showed that students in both studies generally reported high scores in all subscales of the questionnaire after participating in the session with exergames. In particular, students’ scores were above the middle of the scale in the four subscales of the situational interest questionnaire. These scores suggest that exergames have appealing features that attract students’ situational interest [77]. However, this is only a descriptive picture reflecting students’ views after participating in the physical education session with exergames. Thus, no cause-and-effect relationship can be drawn from these results. Future research should examine the effects of exergames on students’ situational interest and compare these effects with the respective effects of other physical activity tasks. Such research should further inform the debate regarding the effects of exergames on students’ situational interests [19]. Indeed, research evidence in this field seems to be mixed. For example, previous findings have suggested that the effects of exergames were higher at the onset of a 4-week intervention compared to other types of physical activity, such as cardiovascular fitness, but these effects declined at the end of the intervention [56,57]. Further research in real life physical education conditions is needed to shed light on the motivational power of exergames.

A strength of this study was that it was conducted in real physical education settings within normal school hours. Previous studies were mainly conducted in laboratory-like settings [49,50], in out-of-school settings [51], or in school settings but in laboratory-like conditions with students participating at an individual level [49,52]. The acute experiments included in these studies were conducted at the class level, in realistic classroom situations, that are inherently social and where the available technological resources may be more constrained than in laboratory settings. Thus, the ecological validity of the results is increased and can be used for establishing sound practical implications.

Thus, from an applied perspective, exergames may be considered an effective means for involving students in physical activity and triggering their executive functions. Moreover, considering that students found that exergames are fun, challenging, and attractive, this means of physical activity may be appropriate for students who are reluctant to participate in physical education. Exergames may also be combined with other cognitively enriched physical education content, such as cognitively challenging physical activity games, to provide students with appropriate and appealing physical education programs targeting both physical and cognitive development [56]. Further, specialists in devising cognitively enriched physical education activities may collaborate with exergame designers with a view to producing innovative exergames that maximize physical and cognitive benefits alike.

## 5. Conclusions

The experiments of these studies showed that exergames can have positive acute effects on students’ executive functions in physical education. Moreover, exergames seem to have appealing features that attract students’ situational interest. The dance exergames used in the present research triggered students’ executive functions by demanding attention, memory, coordination, and speed of action for learning and imitation a sequence of complex movements rather than involving students in automatic replication of simple, segmented movements. Most importantly, these results came from a school-based intervention incorporated into the regular physical education schedule.

A potential limitation of this study was that students’ physical activity during exergames was not measured. Future research should address this limitation by measuring students’ physical activity during exergames and exploring if these amounts of physical activity are associated with the effects of exergames on students’ executive functions. Moreover, a passive control group (i.e., without physical education on testing days) was involved in the first study. However, this was a waiting list control group that received the intervention after the implementation of the intervention with the initial experimental group. Further research is also needed to explore the effects of exergames on students’ executive functions compared to the potential effects of other physical activity tasks. Moreover, considering that this study examined the acute effects of a single physical education session on students’ executive functions, future research should involve larger interventions to examine the long-term effects of exergames in school settings. Such research should also involve multiple tests for measuring all aspects of executive functions, including working memory not measured in this study, and retention measures for evaluating the retention of the effects of exergames on students’ executive functions. The combined effects of exergames with other cognitively enriched physical education content, such as cognitively challenging physical activity games, may be explored. Future research should also involve other types of exergames comparing the effects of exergames with different characteristics on students’ executive functions.

## Figures and Tables

**Table 1:** Descriptive statistics for executive function scores (Study 1).

	Time 1				Time 2				Time 3	
	Experimental		Waitlist Control		Experimental		Waitlist Control		Waitlist Control	
	*M*	*SD*	*M*	*SD*	*M*	*SD*	*M*	*SD*	*M*	*SD*
DF Condition 1	7.44	3.18	7.91	2.14	11.35	3.79	9.08	2.03	11.42	3.14
DF Condition 2	8.58	3.23	7.89	2.35	10.29	3.86	9.17	1.90	11.14	2.16
DF Condition 3	5.29	2.60	4.97	2.06	6.94	2.72	5.58	1.79	7.47	2.16
DF Total	21.32	7.20	20.78	4.89	28.59	8.57	23.83	4.62	30.03	6.42

**Table 2:** Descriptive statistics for situational interest scores.

	Study 1				Study 2	
	Experimental		Waitlist Control		Experimental	
	*M*	*SD*	*M*	*SD*	*M*	*SD*
Total Interest	4.27	0.99	3.98	1.08	4.47	0.93
Instant Enjoyment	4.40	1.05	4.17	1.15	4.41	1.02
Exploration	4.07	1.00	3.71	1.16	4.02	1.10
Novelty	3.98	1.31	3.60	1.32	3.60	1.45

**Table 3:** Descriptive statistics for executive function scores (Study 2).

	Pre-Test		Post-Test	
	*M*	*SD*	*M*	*SD*
DF Condition 1	11.54	4.41	12.82	4.46
DF Condition 2	10.28	4.12	11.33	4.31
DF Condition 3	6.28	3.92	6.79	3.59
DF Total	28.10	9.64	30.95	11.07

## Data Availability

The data underlying the results presented in the study are available on reasonable request from the fourth author (MG; mgoudas@pe.uth.gr).

## References

[1] Staiano A.E., Calvert S.L. (2011). Exergames for physical education courses: Physical, social, and cognitive benefits. Child Dev. Perspect..

[2] Lieberman D. (2006). Dance Games and Other Exergames: What the Research Says.

[3] Papastergiou M. (2009). Exploring the potential of computer and video games for health and physical education: A literature review. Comput. Educ..

[4] Tanaka K., Parker J., Baradov G., Sheehan D., Holash J., Katz L. (2012). A comparison of exergaming interfaces for use in rehabilitation programs and research. Load. J. Can. Game Stud. Assoc..

[5] Konami. https://www.konami.com/en/.

[6] Lieberman D., Vorderer P., Bryant J. (2006). What can we learn from playing interactive games?. Playing Video Games: Motives, Responses, and Consequences.

[7] (2022). Wii. https://en.wikipedia.org/wiki/Wii.

[8] (2022). Wii Balance Board. https://en.wikipedia.org/wiki/Wii_Balance_Board.

[9] Koller J. (2010). This Changes Everything: PlayStation Move. https://blog.playstation.com/2010/06/15/this-changes-everything-playstation-move-available-september-19-2010/.

[10] Rowan D. (2010). Kinect for Xbox 360: The Inside Story of Microsoft’s Secret ‘Project Natal’. Wired.

[11] Canabrava K.L.R., Faria F.R., Lima J.R.P.D., Guedes D.P., Amorim P.R.S. (2018). Energy expenditure and intensity of active video games in children and adolescents. Res. Q. Exerc. Sport.

[12] Graf D.L., Pratt L.V., Hester C.N., Short K.R. (2009). Playing active video games increases energy expenditure in children. Pediatrics.

[13] Verhoeven K., Abeele V.V., Gers B., Seghers J. (2015). Energy expenditure during Xbox Kinect play in early adolescents: The relationship with player mode and game enjoyment. Games Health J..

[14] Fu Y., Burns R.D., Gomes E., Savignac A., Constantino N. (2019). Trends in sedentary behavior, physical activity, and motivation during a classroom-based active video game program. Int. J. Env. Res. Public Health.

[15] Gao Z., Pope Z.C., Lee J.E., Quan M. (2019). Effects of active video games on children’s psychosocial beliefs and school day energy expenditure. J. Clin. Med..

[16] Vernadakis N., Papastergiou M., Zetou E., Antoniou P. (2015). The impact of an exergame-based intervention on children’s fundamental motor skills. Comput. Educ..

[17] Flynn R.M., Staiano A.E., Beyl R., Richert R.A., Wartella E., Calvert S.L. (2018). The influence of active gaming on cardiorespiratory fitness in black and Hispanic youth. J. Sch. Health.

[18] Dos Santos I.K., de Medeiros R.C.D.S.C., de Medeiros J.A., de Almeida-Neto P.F., de Sena D.C.S., Cobucci R.N., Oliveira R.S., Cabral B.G.A.T., Dantas P.M.S. (2021). Active video games for improving mental health and physical fitness—An alternative for children and adolescents during social isolation: An overview. Int. J. Env. Res. Public Health.

[19] Joronen K., Aikasalo A., Suvitie A. (2017). Nonphysical effects of exergames on child and adolescent well-being: A comprehensive systematic review. Scand J. Caring Sci..

[20] Staiano A.E., Beyl R., Guan W., Hendrick C.A., Hsia D.S., Newton R.L. (2018). Home-based exergaming among children with overweight and obesity: A randomized clinical trial. Pediatr. Obes..

[21] Vernadakis N., Zetou E., Derri V., Bebetsos E., Filippou F. (2014). The differences between less fit and overweight children on enjoyment of exergames, other physical activity and sedentary behaviours. Procedia Soc. Behav. Sci..

[22] Diamond A. (2013). Executive functions. Annu. Rev. Psychol..

[23] Diamond A. (2015). Effects of physical exercise on executive functions: Going beyond simply moving to moving with thought. Annu. Sport. Med. Res..

[24] Blair C., Raver C. (2015). School readiness and self-regulation: A developmental psychobiological approach. Annu. Rev. Psychol..

[25] Mazzocco M.M., Kover S.T. (2007). A longitudinal assessment of executive function skills and their association with math performance. Child. Neuropsychol..

[26] Schmidt M., Egger F., Benzing V., Jäger K., Conzelmann A., Roebers C.M., Pesce C. (2017). Disentangling the relationship between children’s motor ability, executive function and academic achievement. PLoS ONE.

[27] Roebers C.M. (2017). Executive function and metacognition: Towards a unifying framework of cognitive self-regulation. Dev. Rev..

[28] Kolovelonis A., Goudas M., Dermitzaki I. (2010). Self-regulated learning of a motor skill through emulation and self-control levels in a physical education setting. J. Appl. Sport Psychol..

[29] Kolovelonis A., Goudas M., Dermitzaki I., Kitsantas A. (2013). Self-regulated learning and performance calibration among elementary physical education students. Eur. J. Psychol. Educ..

[30] Pesce C., Vazou S., Benzing V., Alvarez-Bueno C., Anzeneder S., Mavilidi M., Leone L., Schmidt M. (2021). Effects of chronic physical activity on cognition across the lifespan: A systematic meta-review of randomized controlled trials and realist synthesis of contextualized mechanisms. Int. Rev. Sport Exerc. Psychol..

[31] Vazou S., Pesce C., Lakes K., Smiley-Oyen A. (2019). More than one road leads to Rome: A narrative review and meta-analysis of physical activity intervention effects on cognition in youth. Int. J. Sport Exerc. Psychol..

[32] Gentile A., Boca S., Şahin F.N., Güler Ö., Pajaujiene S., Indriuniene V., Demetriou Y., Sturm D., Gómez-López M., Bianco A. (2020). The effect of an enriched sport program on children’s executive functions: The ESA Program. Front. Psychol..

[33] Alesi M., Bianco A., Luppina G., Palma A., Pepi A. (2016). Improving children’s coordinative skills and executive functions: The effects of a football exercise program. Percept. Mot. Ski..

[34] Singh A.S., Saliasi E., van den Berg V., Uijtdewilligen L., de Groot R., Jolles J., Andersen L.B., Bailey R., Chang Y.-K., Diamond A. (2019). Effects of physical activity interventions on cognitive and academic performance in children and adolescents: A novel combination of a systematic review and recommendations from an expert panel. Br. J. Sport. Med..

[35] Wassenaar T.M., Williamson W., Johansen-Berg H., Dawes H., Roberts N., Foster C., Sexton C.E. (2020). A critical evaluation of systematic reviews assessing the effect of chronic physical activity on academic achievement, cognition and the brain in children and adolescents: A systematic review. Int. J. Behav. Nutr. Phys. Act..

[36] Paschen L., Lehmann T., Kehne M., Baumeister J. (2019). Effects of acute physical exercise with low and high cognitive demands on executive functions in children: A systematic review. Pediatr. Exerc. Sci..

[37] Parlebas P. (2020). The universals of games and sports. Front. Psychol..

[38] Lubans D.R., Leahy A.A., Mavilidi M.F., Valkenborghs S.R. (2022). Physical activity, fitness, and executive functions in youth: Ef-fects, moderators, and mechanisms. Curr. Top. Behav. Neurosci..

[39] Diamond A., Ling D., Novick J., Bunting M., Dougherty M., Engle R. (2020). Review of the evidence on, and fundamental questions about, efforts to improve executive functions, including working memory. Cognitive and Working Memory Training: Perspectives from Psychology, Neuroscience, and Human Development.

[40] Tomporowski P., McCullick B., Pesce C. (2015). Enhancing Children’s Cognition with Physical Activity Games.

[41] Pesce C., Masci I., Marchetti R., Vazou S., Sääkslahti A., Tomporowski P.D. (2016). Deliberate play and preparation jointly benefit motor and cognitive development: Mediated and moderated effects. Front. Psychol..

[42] Kolovelonis A., Goudas M. (2022). Exploring the effects of three different types of cognitively challenging physical activity games on students’ executive functions and situational interest in physical education. Cogent Educ..

[43] Kolovelonis A., Goudas M. (2022). Acute enhancement of executive functions through cognitively challenging physical activity games in elementary physical education. Eur Phy Educ Rev..

[44] Kolovelonis A., Goudas M. (2022). The effects of cognitively challenging physical activity games versus health-related fitness activities on students’ executive functions and situational interest in physical education. A Group-Randomized Control. Trial.

[45] Kolovelonis A., Pesce C., Goudas M. (2022). The effects of a cognitively challenging physical activity intervention on school children’s executive functions and motivational regulations. Int. J. Environ. Res. Public Health..

[46] Eggenberger P., Wolf M., Schumann M., de Bruin E.D. (2016). Exergame and balance training modulate prefrontal brain activity during walking and enhance executive function in older adults. Front. Aging Neurosci..

[47] Huang K.T. (2020). Exergaming executive functions: An immersive virtual reality-based cognitive training for adults aged 50 and older. Cyberpsychol. Behav. Soc. Netw..

[48] Kayama H., Okamoto K., Nishiguchi S., Yamada M., Kuroda T., Aoyama T. (2014). Effect of a Kinect-based exercise game on improving executive cognitive performance in community-dwelling elderly: Case control study. J. Med. Internet Res..

[49] Best J.R. (2012). Exergaming immediately enhances children’s executive function. Dev. Psychol..

[50] Flynn R.M., Richert R.A. (2018). Cognitive, not physical, engagement in video gaming influences executive functioning. J. Cogn. Dev..

[51] Flynn R.M., Richert R.A., Staiano A.E., Wartella E., Calvert S.L. (2014). Effects of exergame play on EF in children and adolescents at a summer camp for low-income youth. J. Educ. Dev. Psychol..

[52] Benzing V., Heinks T., Eggenberger N., Schmidt M. (2016). Acute cognitively engaging exergame-based physical activity enhances executive functions in adolescents. PLoS ONE.

[53] Roure C., Pasco D. (2018). Exploring situational interest sources in the French physical education context. Eur. Phy. Educ. Rev..

[54] Chen S., Sun H., Zhu X., Chen A. (2014). Relationship between motivation and learning in physical education and after-school physical activity. Res. Q. Exerc. Sport.

[55] Roure C., Pasco D., Benoît N., Deldicque L. (2020). Impact of a design-based bike exergame on young adults’ physical activity metrics and situational interest. Res. Q. Exerc. Sport.

[56] Sun H. (2012). Exergaming impact on physical activity and interest in elementary school children. Res. Q. Exerc. Sport.

[57] Sun H. (2013). Impact of exergames on physical activity and motivation in elementary school students: A follow-up study. J. Sport Health Sci..

[58] Sun H., Gao Y. (2016). Impact of an active educational video game on children’s motivation, science knowledge, and physical activity. J. Sport Health Sci..

[59] UNESCO (2017). Kazan Action Plan. http://unesdoc.unesco.org/images/0025/002527/252725e.pdf.

[60] Pesce C., Faigenbaum A., Goudas M., Tomporowski P., Meeusen R., Schaefer S., Tomporowski P., Bailey R. (2018). Coupling our plough of thoughtful moving to the star of children’s right to play: From neuroscience to multi-sectoral promotion. Physical Activity and Educational Achievement: Insights from Exercise Neuroscience.

[61] Delis D.C., Kaplan E., Kramer J.H. (2001). Delis-Kaplan Executive Function System (D-KEFS).

[62] Roure C., Pasco D., Kermarrec G. (2016). Validation de l’échelle française mesurant l’intérêt en situation, en éducation physique. Can. J. Behav. Sci. Rev. Can. Des. Sci. Comport..

[63] Ubisoft (2015). Just Dance. https://www.ubisoft.com/en-gb/game/just-dance/2015.

[64] Pan European Game Information (PEGI). https://pegi.info/.

[65] Fandom Just Dance 2015. https://justdance.fandom.com/wiki/Just_Dance_2015.

[66] Flynn R.M., Colon N. (2016). Solitary active videogame play improves executive functioning more than collaborative play for children with special needs. Games Health J..

[67] Wikipedia (2022). Just Dance 2015. https://en.wikipedia.org/wiki/Just_Dance_2015.

[68] Hwang J., Hillman C.H., Lee I.M., Fernandez A.M., Lu A.S. (2021). Comparison of inhibitory control after acute bouts of exergaming between children with obesity and their normal-weight peers. Games Health J..

[69] Staiano A.E., Abraham A.A., Calvert S.L. (2012). Competitive versus cooperative exergame play for African American adolescents’ executive function skills: Short-term effects in a long-term training intervention. Dev. Psychol..

[70] Marker A.M., Staiano A.E. (2015). Better together: Outcomes of cooperation versus competition in social exergaming. Games Health J..

[71] Fandom Happy. https://justdance.fandom.com/wiki/Happy.

[72] Fandom Love Me Again. https://justdance.fandom.com/wiki/Love_Me_Again.

[73] Benzing V., Chang Y.K., Schmidt M. (2018). Acute physical activity enhances executive functions in children with ADHD. Sci. Rep..

[74] Anderson-Hanley C., Tureck K., Schneiderman R.L. (2011). Autism and exergaming: Effects on repetitive behaviors and cognition. Psychol. Res. Behav. Manag..

[75] Cohen J. (1988). Statistical Power Analysis for the Behavioral Sciences.

[76] Benzing V., Schmidt M. (2019). The effect of exergaming on executive functions in children with ADHD: A randomized clinical trial. Scand. J. Med. Sci. Sport..

[77] Chen A., Ennis C.D., Martin R., Sun H., Hogan S.A. (2006). Situational interest: A curriculum component enhancing motivation to learn. New Developments in Learning Research.

